# Lesion-specific circular RNA and mRNA signatures in secondary progressive multiple sclerosis

**DOI:** 10.1186/s13041-026-01333-y

**Published:** 2026-07-22

**Authors:** Yulan Gao, Konii Takenaka, Kristina Santucci, Grace Lindner, Yuning Cheng, Si-Mei Xu, Michael Janitz

**Affiliations:** School of Biotechnology and Biomolecular Sciences, University of New South Wales, Sydney, NSW 2052 Australia

## Abstract

Circular RNAs (circRNAs) are single-stranded RNAs with a covalently closed loop structure which have been increasingly recognised as stable and functionally relevant regulators of gene expression via sponging miRNAs. Recently, studies have revealed their roles in human diseases, subsequently emerging as potential biomarkers. Multiple sclerosis (MS) is an inflammatory, neurodegenerative, and immune-related disease of the central nervous system characterised by distinct lesion types that reflect different stages of brain tissue injury and repair. Our study conducted a comprehensive transcriptomic analysis of circRNAs and linear mRNAs across secondary progressive MS (SPMS) lesion types, including normal-appearing white matter, active lesions, chronic active lesions, inactive lesions, and control white matter tissue. Using RNA-seq data and bioinformatics-based analysis, we identified lesion-specific circRNAs and linear transcripts signatures and visualised their expression patterns across samples using row-scaled heatmaps. Functional enrichment analysis revealed that dysregulated circRNAs were associated with synaptic organisation, intracellular signalling, immune responses, and chromatin regulation. To explore potential regulatory mechanisms, we constructed hub genes networks for circQSER1-hsa-miR-6891-5p/hsa-miR-6758-5p-*SCD*, circFMN2-hsa-miR-4728-5p-*SCD* axes from down-regulated active lesion type, circCCDC7-hsa-miR-4659b-3p-*HBEGF* axes from chronic active lesion type for circRNA-miRNA-mRNA. Detailed visualisation of the circRNAs that were identified to be involved in the axes demonstrated extensive isoform diversity and circularisation events within key loci. Our results highlight that circRNAs-associated networks exhibit lesion-specific expression and structural diversity in SPMS and may participate in MS-associated biological processes. This study provides a deeper understanding of circRNAs dysregulation across lesion types and uncovers potential candidate targets and candidate regulatory axes.

**Supplementary Information:**

The online version contains supplementary material available at https://doi.org/10.1186/s13041-026-01333-y.

## Introduction

Multiple sclerosis (MS) is a chronic, immune-mediated disease which has been revealed to have increasing global cases and consistently high mortality rates due to lack of diagnostic capacity, particularly in low- and middle-income countries [1]. It is characterised by neuroinflammation, demyelination, and neurodegeneration that disrupts the normal flow of signals along nerve fibres. Although the exact cause of MS remains unclear, current evidence suggests a complex interaction between genetic susceptibility, environmental triggers, and abnormal immune responses. Over time, these processes lead to the attack of myelin by immune cells, forming scar tissues known as plaques or lesions, and the blocking of nerve transmission by these plaques or lesions. Symptoms include numbness, muscle spasms, vision issues, as well as cognitive problems, which differ across individual patients [2].

MS disease progression typically starts with relapsing-remitting MS (RRMS), marked by relapses followed by period of partial recovery also known as remission [3, 4]. However, for many patients, the disease develops into secondary progressive MS (SPMS) which is characterised by an accumulation of lesions, fewer relapses, and progressively worsening neurological deficits and disability. A hallmark of SPMS is the presence of different lesion types within the white matter of the brain. These lesions represent varying stages of inflammation, demyelination, and repair. Normal appearing white matter (NAWM), which leads to pathological states, shows subtle demyelination, axonal transection, early microglial activation, and leakage of blood-brain barrier [5]. The lesion can then develop into active lesions (AL) where myelin integrity is severely reduced with high inflammation and infiltration of immune cells, and extensive demyelination. The AL are rich in activated microglia and macrophages that induce inflammatory cytokines, therefore, highlighting the intense immune-driven phase of MS pathology [6]. Remyelinating lesions (RL) can develop when the human body attempts to protect and repair the damage by partially restoring myelin [7–9]. The repair process is supported by oligodendrocyte precursor cells which differentiate and recoat the axons with a new but thin myelin. The repair process is more active at the early stage of SPMS and becomes sparser at later stages due to the reduced number and impairment of oligodendrocytes precursor cells [10]. Unlike acute inflammation that reveals massive and quick damage, chronic active (CA) lesions show continuous, smouldering inflammation at the edge of lesions [11, 12]. The rim lesions slowly expand over time, causing gradual tissue destruction, failed myelin repair and axonal loss. As the pathogenesis of MS develops into inactive lesions (IL), sharply defined scars with demyelinated axons and glial scarring, as well as the immune cells have mostly been cleared out for inactively inflamed [13]. These tissues show signs of long-term structural destructions, hence, the low capacity for repair indicates the end stage of damage. These lesion types outline the complexity of pathogenesis of MS, thus understanding the progression is essential for developing therapeutic strategies, yet the biological drivers that control the transition to SPMS remain poorly defined.

A growing interest in the role of circRNAs in neurological diseases has been evident in the recent years. CircRNAs are a class of endogenous RNA molecules formed through a back-splicing event, where a downstream splice donor ligates to upstream splice acceptor, creating a covalently closed loop [14–16]. The lack of a 5’ cap and 3’ poly(A) tail prevents circRNAs from being degraded, which allow them to be highly stable and evolutionarily conserved [17, 18]. CircRNAs have been found to be enriched in brain tissue and playing a significant regulatory role during disease progression [14, 19]. The interaction between circRNAs and RNA-binding proteins promote formation of complexes that regulate gene expression. Ashwal-Fluss and Meyer [16] identified that circMbl sequesters the muscleblind splicing factor (Mbl) in a negative autoregulatory feedback loop controlling alternative splicing of its own host-pre-mRNA. In neurological disease, a well discovered circRNA names as cerebellar degeneration-related 1 antisense RNA (CDR1as), also known as ciRS-7, is most abundantly expressed in the human brain [20]. By regulating the target miR-7, CDR1as functionally impact on sensorimotor gating and synaptic transmission in neurons. One of the roles is interacting with microRNAs (miRNAs), which are small non-coding RNAs (21–23 bps), via base pairing with miRNA response element (MRE) [21]. circRNAs and mRNAs can function as competing endogenous RNAs, where they can bind to miRNAs and their expression and be modulated during interactions [22]. When circRNAs that contain MREs and act as miRNA sponges, the availability of miRNAs is reduced, and eventually, the miRNA-target mRNAs are rescued for expressions. Studies by Cardamone and Paraboschi [23] and Iparraguirre and Muñoz-Culla [24] published in 2017 initiated the investigation of circRNAs in MS, particularly RRMS. Exonic circular RNA derived from the Gasdermin B gene (*GSDMB*) was identified in the most of brains regions from RRMS patients, as well as showing an up-regulating pattern in the serum and cerebrospinal fluid [23]. Iparraguirre and Muñoz-Culla [24] carried a circRNA profiling study in peripheral blood, and revealed that approximately 80% of expressed circRNAs were down-regulated in the samples. In particular, two down-regulated circRNA isoforms originating from the Annexin A2 gene (*ANXA2*) were identified as isoforms with potential correlation with blood-brain barrier. Additionally, the circRNA Sphingomyelin synthase-related protein 1 (*SAMD8*) was one of the few that have been predicted from SPMS samples. Mycko and Zurawska [25] constructed networks formed by hsa_circRNA_001896 and hsa_circ101145, targeting ten miRNAs targeting three mRNAs. These networks were suggested to impact on such as oxidative stress, blood-brain barrier permeability, and microglia function for MS pathogenesis. Overall, the circRNA-miRNA-mRNA networks in SPMS remains elusive and their relative biological processes requires in-depth investigation.

Given the layered and dynamic pathology of MS lesions, it is crucial to understand the mechanisms and the roles of circRNAs and their downstream networks alterations across different lesion types. The wide-use of RNA-sequencing (RNA-seq) technology has enabled the development of bioinformatics tools, allowing the analysis of an immense number of transcriptomic datasets accurately and at scale [26–29]. Various forms of RNA can be detected and predicted for potential biological meanings, which provide researchers with a rapid and systematic scan of the RNA molecules’ functions in the disease. Therefore, this study aims to identify circRNAs across lesion types, determine associated miRNAs and mRNA targets, and construct circRNA-miRNA-mRNA/hub genes networks that reflect the biological significance within the MS brain. By analysing RNA-seq data, we aimed to explore the participation of the molecules in inflammation, degeneration, and repair, and to provide new insights into the molecular landscape for the progression of SPMS.

## Materials and methods

### Data retrieval

The human post-mortem brain tissue was collected from UK Multiple Sclerosis Society Tissue Bank for subsequential lesion classification and RNA-sequencing. Detailed generation of raw paired-end RNA-seq datasets can be referred to the paper of origin by Elkjaer and Frisch [2] A total of 19 normal appearing white matter (NAWM), 18 active lesion (AL), 13 inactive lesion (IL), 17 chronic active lesion (CA), six remyelinating lesion (RL) and 25 control white matter RNA-seq samples were initially retrieved from GEO database (GSE138614) [2]. Control white matter samples were post-mortem white matter from neurological normal donors with no MS diagnosis and no white matter pathology on histological examination.

### RNA-seq data quality check and filtration

Quality check was performed on all raw RNA-seq samples using FastQC v0.11.9, as well as on the samples that were trimmed by Trimmomatic v0.39 [30, 31]. All trimmed samples were aligned against the human annotation reference GENCODE v48 using STAR v2.7.9a [32, 33]. Filtration of the sequencing samples was completed, where samples that exhibited a GC content of 40–60% from the FastQC tool and passed at least 80% STAR unique alignment rate proceeded to downstream analysis. Therefore, 17 NAWM, six AL, eight IL, 13 CA and 23 control paired-end samples were selected for this study. The remyelinating lesion dataset was excluded from the datasets as only one paired-end sample passed the filtering criteria.

### Identification of circRNAs and mRNAs

To identify the circRNAs from the filtered RNA-seq samples, two parallel analytical pipelines were used to capture a robust landscape of circRNAs. After STAR alignment, CIRCexplorer2 *-parse* and *-annotate* modes were used to parse junction reads from the *starChimeric.out.junction* files and annotate the inferred circRNAs, respectively [34]. Similarly, the trimmed sequencing reads were aligned using the BWA tool with the genome sequence from UCSC hg38 [35]. The output *sam* files were sent to CircRNA Identifier 2 (CIRI2) (CIRI v2.0.6) for identification of circRNAs [36, 37]. For each sample, common circRNAs detected from both pipelines were identified by both pipelines were selected for downstream analysis. The number of circRNAs identified across the lesion types and controls samples were constructed into Venn diagram by the Venn v1.12 package on Rstudio [38, 39].

Additionally, linear transcripts were identified, quantified and annotated using HISAT2 v2.2.0 alignment tool [40] followed by StringTie v1.3.4d [41], which also generated gene count matrix through its preDE.py3.

### Differentially expressed circRNAs and linear transcripts

A differential expression (DE) analysis of circRNAs and linear transcripts across each lesion type and control samples was performed in R v4.3.1 and packages were managed by BiocManager v1.30.25. All individual circRNA count files were collected by edgeR v4.0.16 *readDGE* to make into DEGlist and filtered using using filterByExpr [42, 43]. *limma*’s voom took the raw junction read counts and the effective library sizes, which were adjusted by the Trimmed Mean of M-values normalisation (TMM) method, to transform into log counts per million (CPM) and reduce variance [44]. The ggplot2 v3.5.1 package was used for volcano plots to visualise DE circRNAs and linear transcripts (|logFC| > 0.5, adj.P.Val < 0.1) [45]. The use of adjusted p-value was achieved by the Benjamini-Hochberg method to improve the statistical significance and control the false discovery rate across the samples. The expression of DE circRNAs and linear transcripts across the samples was visualised with the pheatmap v1.0.13 package [46]. The cross-condition heatmap displayed row-scaled Z-scores of logFC values across the four lesion-versus-control contrasts, which represented standard deviations from the mean expression pattern of each gene across the four lesion types.

### Gene ontology enrichment and Kyoto Encyclopedia of Genes and Genomes analysis

Gene ontology (GO) enrichment analysis which comprised biological process (BP), cellular component (CC), molecular function (MF) was performed independently for all DE circRNAs across the four lesion types using clusterProfiler v4.10.1 [47]. A Kyoto Encyclopaedia of Genes and Genomes pathway analysis was also completed with the detected DE circRNAs. Enriched terms and pathways in the GO and KEGG analysis were grouped into a *treeplot* for comprehensive visualisation when there were at least five terms or pathways were enriched, otherwise a *dotplot* was used for simple visualisation. The Benjamini-Hochberg method was applied to all enrichment analysis to adjust the p-value (p.adjust < 0.05).

### Prediction of circRNA-miRNA-mRNA networks

CircRNA-miRNA pairs were predicted by Circr, a tool which integrates three miRNA-target prediction tools including miRanda, RNAhybrid and TargetScan [48]. Only circRNAs that exhibited at least seven miRNA response elements(MREs) were selected, since miRNAs need seed sequences (6–8 bp long) to bind to the target sequence located on the sequences. The predicted miRNA lists were further analysed for miRNA-mRNA pairs using miRWalk and MultiMiR v2.4.0 [21, 49, 50]. Common miRNA-mRNA pairs detected by both tools were selected for circRNA-miRNA-mRNA network construction.

### Prediction of the hub genes and circRNA-miRNA-hub gene networks

From the DE mRNAs, hub genes were identified based on the protein-protein interaction (PPI) predicted by STRING (interaction score with highest confidence > 0.9). Of note, hub genes are genes that interact with other genes with high connectivity degression in terms of co-expression. Predicted PPI networks were visualised in Cytoscape v3.10.3, with the cytoHubba plug-in offering several topological algorithms including maximal clique centrality (MCC) and maximum neighbourhood component (MNC) for identifying key genes within complex networks via ranking nodes from the property [51, 52]. Prediction of miRNA-hub gene pairs was accomplished using the results from miRWalk and MultiMiR. To evaluate white matter-specific expression of miRNAs incorporated in the ceRNA networks, average expression values were retrieved from the tool miRNATissueAtlas 2025 [53], selecting *Brain* as the organ system and *Brain* as the tissue. Average expression values (avg_expression) ± standard deviation (stddev_expression) were calculated based on their formula including a specific evaluation of tissue specificity index, organ, reads per million mapped reads (RPMM) from the samples.

### GEO database validation and ROC curves

Other RNA-seq MS sample datasets, namely GSE255290 (*n* = 10) and GSE224377 (*n* = 36), were also utilised to validate the expression of the hub genes that were predictively involved in the miRNA-hub genes pairs. A receiver operating characteristic (ROC) curve analysis was performed to evaluate the importance of hub genes in MS progression in Prism v10 (https://www.graphpad.com).

### Visualisation of the circRNAs that are involved in the networks

To further understand the circRNAs’ structures, we used CIRI2, CIRI-AS, CIRI-full and CIRI-vis pipelines which could display circRNAs isoforms from particular MS lesion types. Since CIRI-vis outputs are in a PDF format, imagemagick v7.1.2-9 and ghostscript v10.06.0 were used to convert the files into a PNG format with high image resolution [54, 55].

## Results

After quality check, the filtered RNA-seq MS dataset included 17 NAWM, six AL, 13 CA, eight IL and 23 control samples which used for the downstream analysis, including the identification of circRNAs and linear transcripts as well as the circRNA-miRNA-mRNA/hub genes networks construction. Different functions and gene expression were analysed depending on the types of lesions.

### Identification of DE circRNAs and DE linear transcripts

**Fig. 1:**
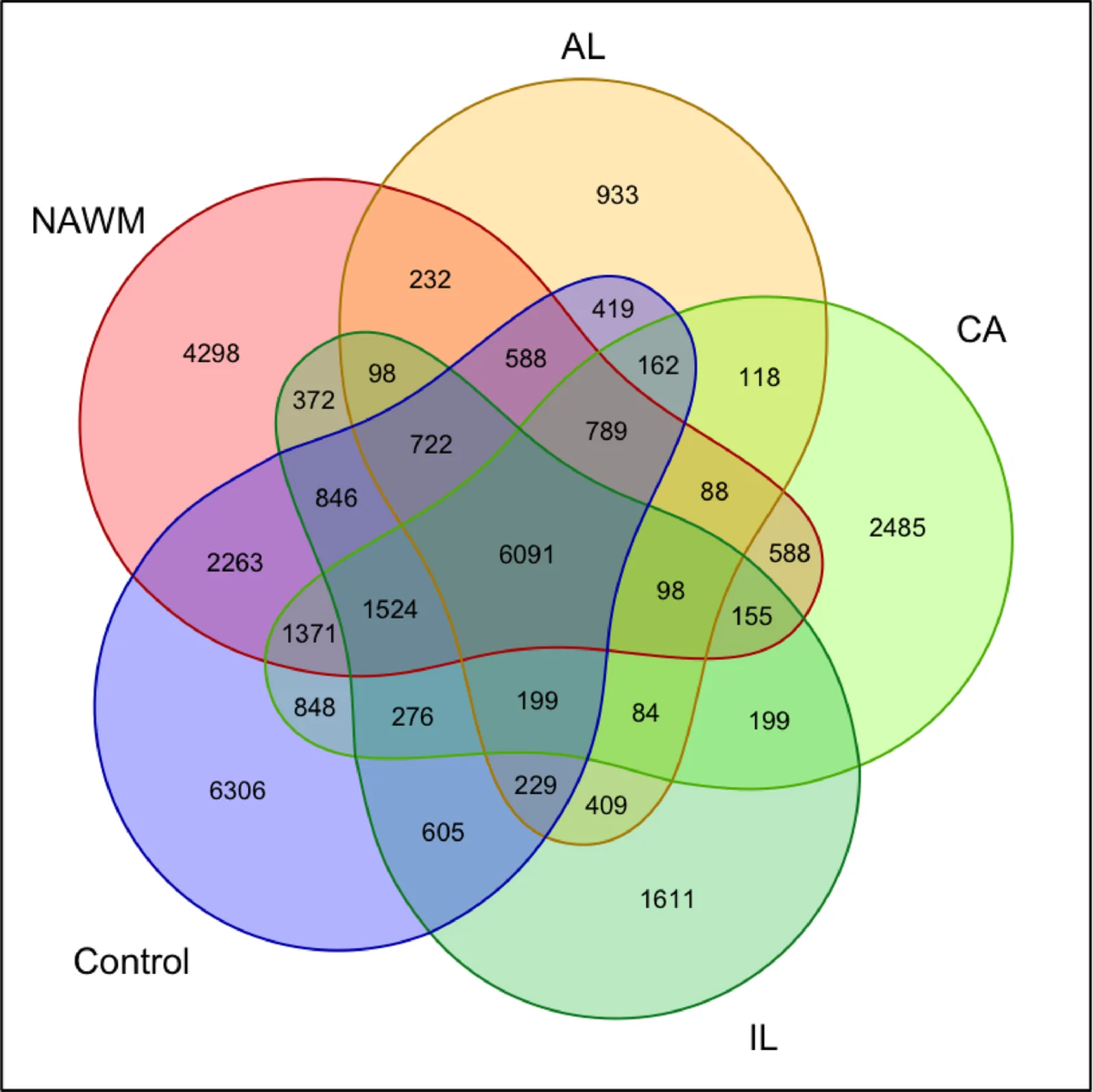
Venn diagram of circRNAs identified across four lesion types and control samples. Normal appearing white matter (NAWM); Active lesion (AL); Chronic active (CA); Inactive lesion (IL); Control (healthy control samples)

6091 common circRNAs were identified across all lesion types and control samples (Fig. 1). Each lesion exhibited uniquely expressed circRNAs, including 4,298 in NAWM, 933 in AL, 2,485 in CA, 1,611 in IL and 6306 in control. Detailed intersected circRNAs with their coordinates are listed in Table S1.

**Fig. 2:**
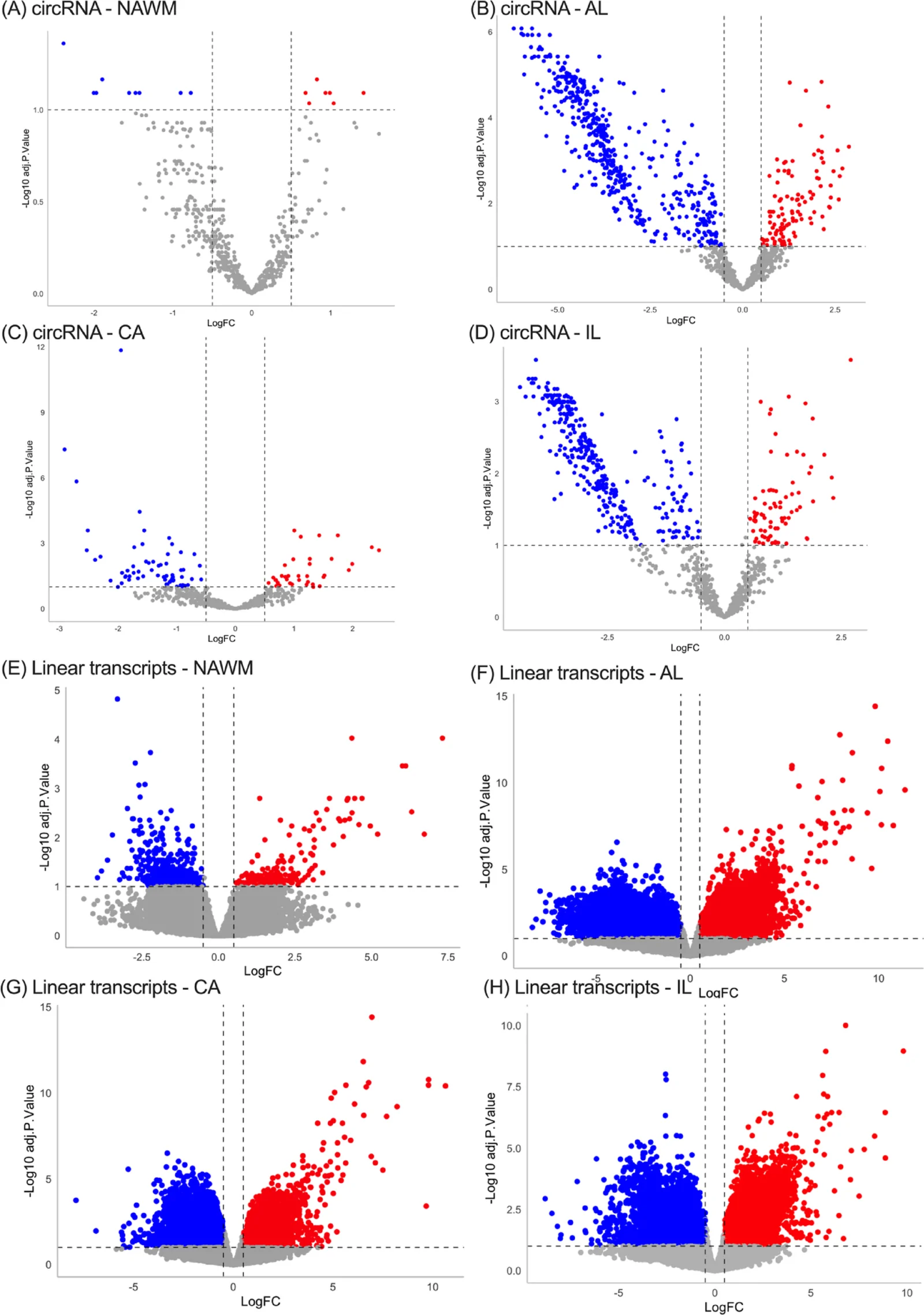
Volcano plots of DE circRNAs and linear transcripts. Panel **A**–**D** displayed DE circRNAs and panels **E**–**H** displayed DE linear transcripts for each lesion condition as labelled in the panel. Each panel plotted |log_2_FC| on the x-axis against -log10(adjusted p.value) on the y-axis. Blue dots: significantly down-regulated transcripts; red dots: significantly up-regulated transcripts; grey dots: non-significant transcripts

**Fig. 3:**
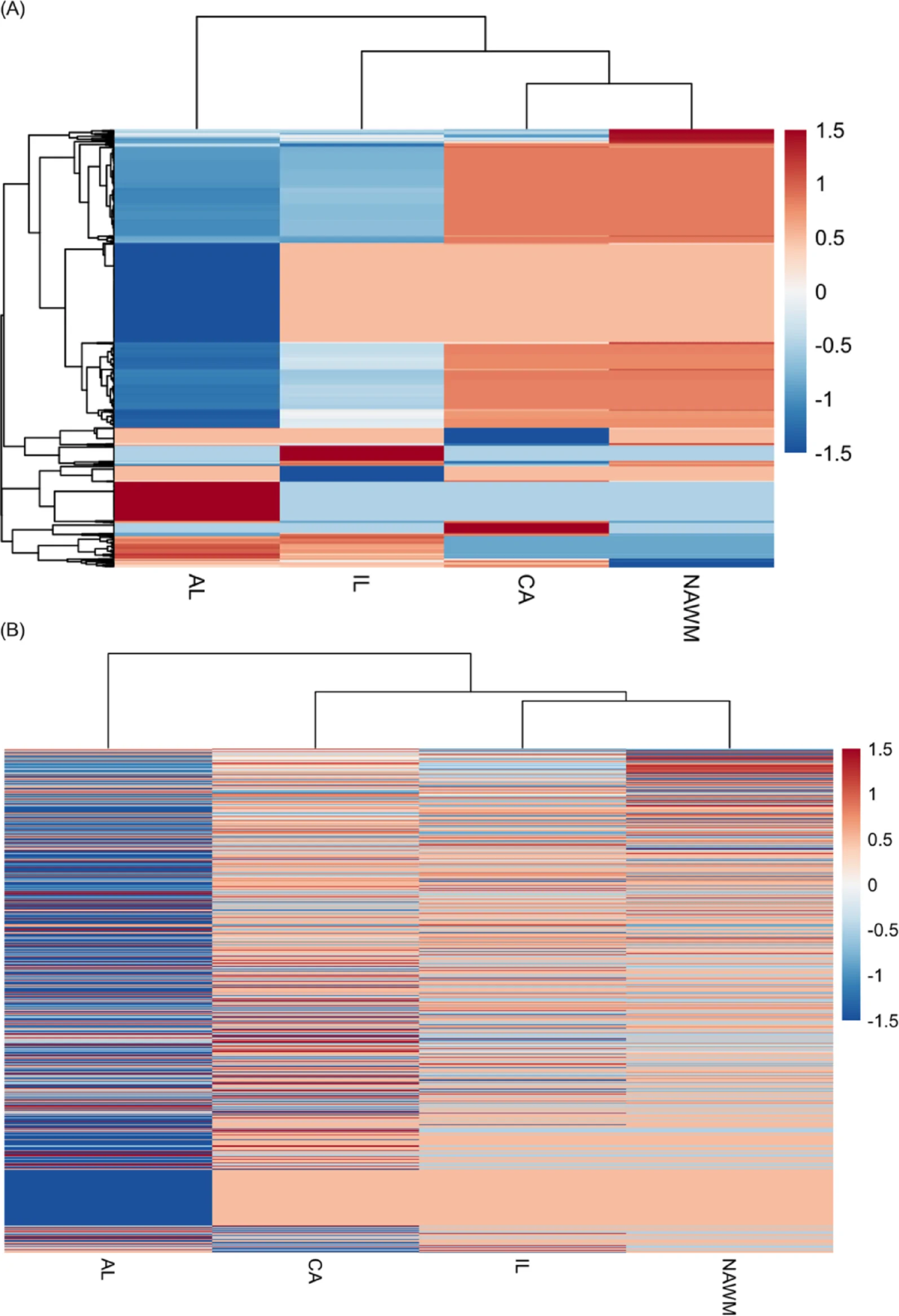
Heatmap of DE circRNAs and linear transcripts. Relative expression changes of circRNAs (**A**) and linear transcripts (**B**) were shown as row-scaled Z-scores of logFC. The Z-score scale spanned − 1.5 to 1.5, corresponding to a colour gradient from blue (lower than the mean) to red (higher than the mean). Columns (AL/CA/IL/NAWM) are clustered by similarity, giving the dendrogram on top

Differential expression analysis of circRNAs and linear transcripts was performed for each MS lesion stage versus control white matter (Fig. 2). Among circRNAs, the NAWM versus control comparison identified the least DE transcripts, with scattered blue and red points located near the low significance threshold. In contrast, AL versus control comparison revealed the most extensive dysregulated circRNAs, characterised by a prominent cluster of down-regulated circRNAs, smaller population of up-regulated transcripts, and high statistical significance. AL condition produced the densest cloud of DE linear transcripts, reflecting the widespread dysregulation. The CA and IL versus control comparisons showed relatively moderate DE circRNAs and linear transcripts without extreme logFC magnitudes. Figure 3 showed the logFC patterns for DE circRNAs or linear transcripts from each lesion versus control samples. A lower number of DE features is observed in the DE circRNAs in comparison to DE linear transcripts, indicated through a lower number of rows. NAWM and CA lesions had the most similar DE circRNAs patterns while NAWM and IL lesions had the most similar patterns in DE linear transcription. The AL heatmap pattern is distinct from the other three lesions from the hierarchical clustering of columns for both DE circRNAs and linear transcripts.

### GO terms and KEGG pathways

**Fig. 4:**
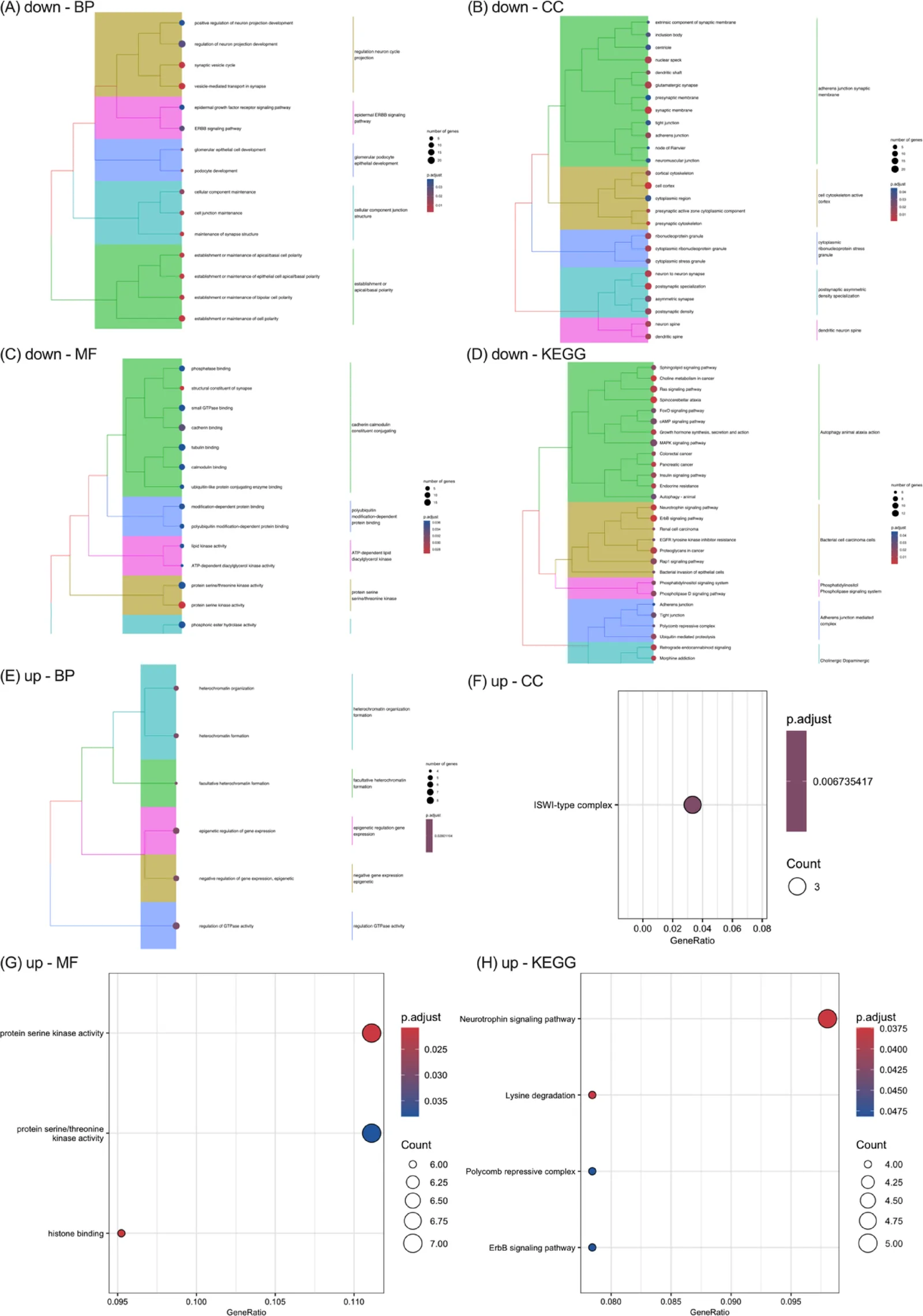
GO terms and KEGG pathways analysis for AL. Panel **A**–**D** showed enriched biological process (BP), cellular component (CC), molecular function (MF), KEGG pathway for down-regulated circRNAs. Panel E – H showed the enriched functional GO terms and pathways in the same order for up-regulated circRNAs. The significance of p.adjust was represented in the colour gradient as indicated on each bar. Grouped trees were generated when there were more than five elements enriched, or dot plots shown for the number of genes associated with each term and the statistical significance of enrichment

**Fig. 5:**
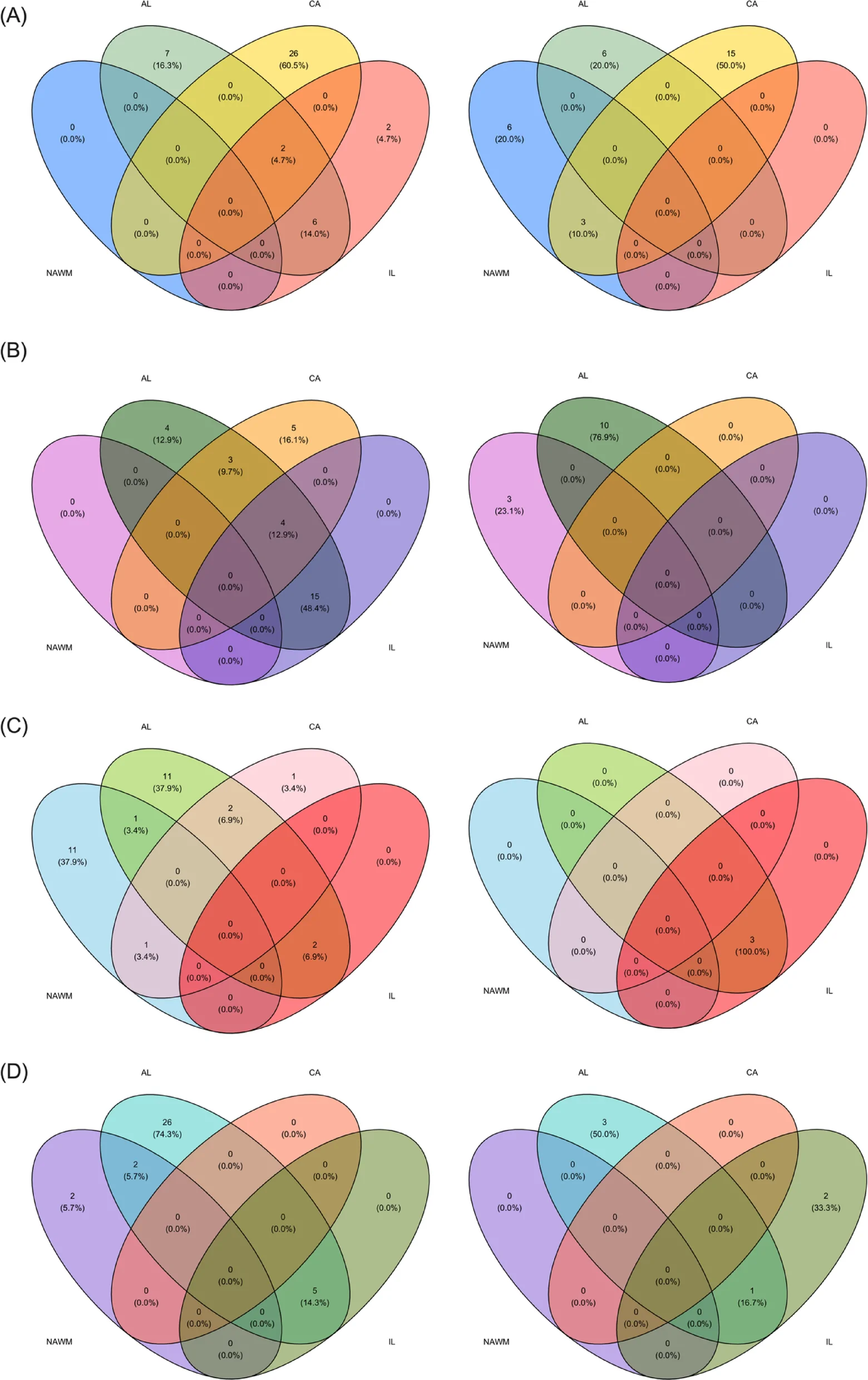
The common GO terms and KEGG pathways enriched by the DE circRNAs. Enriched GO terms were in order of BP, CC, MF (**A**–**C**), and KEGG pathways in panel **D**. Terms and pathways for down-regulated circRNAs were on the left and up-regulated circRNAs were on the right

Host genes of circRNAs were analysed to determine the potential functions of the DE circRNAs via enrichment analysis for all four lesion types (Figs. 4 and 5). Host gene names of DE circRNAs were submitted for enrichment analysis, including 481 down-regulated circRNAs and 111 up-regulated circRNAs from AL versus control. The analysis showed that in AL, all three types of GO terms (BP, CC, MF) and KEGG pathways were enriched (Fig. 4). Figure S1–S3 included the enriched terms and pathways for NAWM, IL and CA. Less dysregulated circRNAs from these three lesion types resulted in less enriched GO terms and pathways. Clustered terms for host genes of down-regulated circRNAs significantly reflected dysregulation of pathways including neuronal regulations, synaptic signalling and structure, cytoskeletal and microtubule-associated functions. KEGG pathways for the down-regulated circRNAs were majorly grouped into autophagic dysregulaion, where a large number of proteins and intracellular signalling pathways were involved in damaging the cell health and homeostasis. Pathways for neuronal repair and structural maintenance were associated with down-regulated circRNAs. Host genes of up-regulated circRNAs were enriched strongly in chromatin organisation, epigenetic regulation and phosphorylation cascades. Two major biological themes in the KEGG pathways were confirmed, including epigenetic and chromatin regulation, and cellular signalling pathways.

### Prediction of circRNA-miRNA-mRNA/hub genes networks

**Fig. 6:**
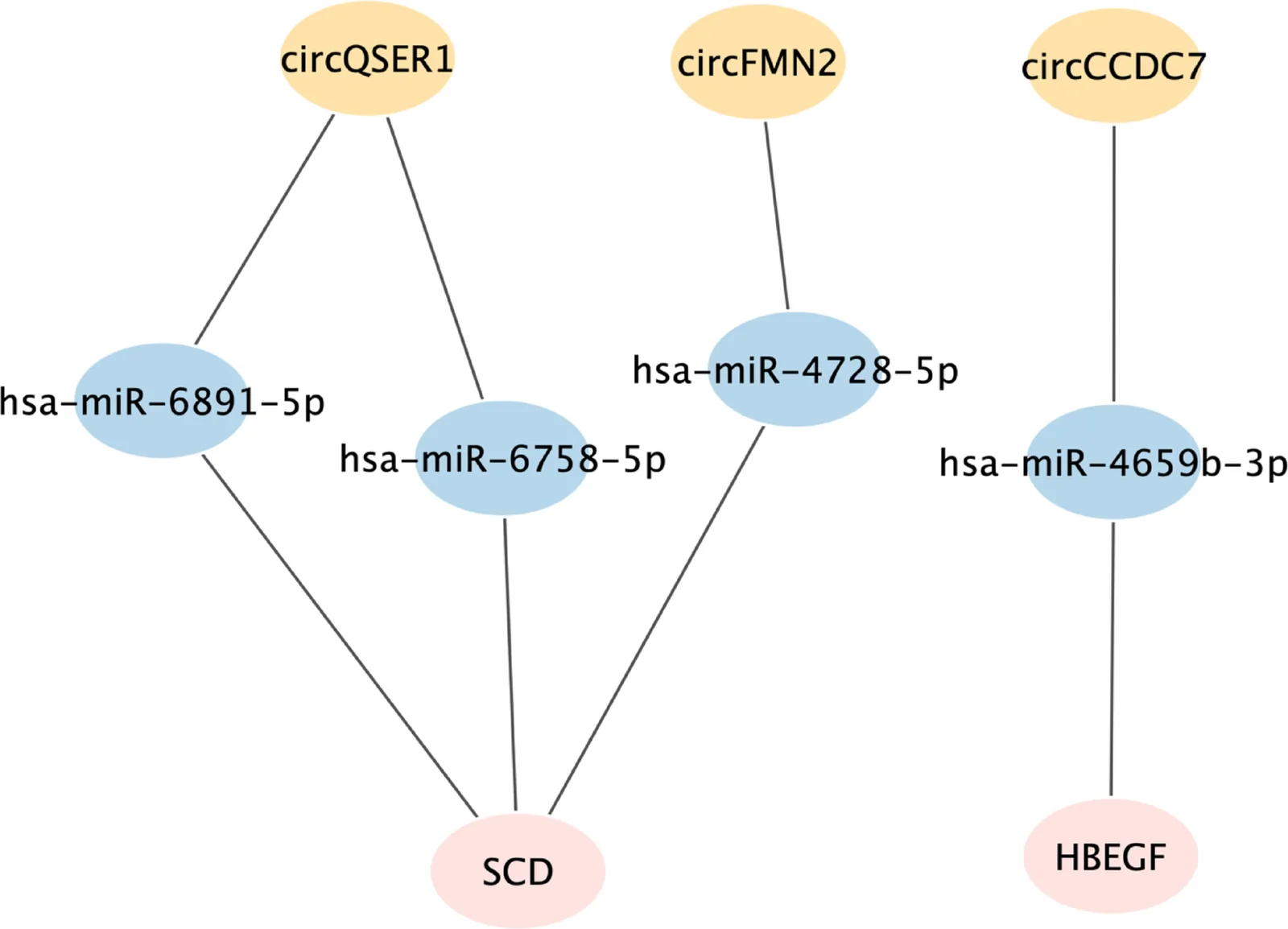
CircRNA-miRNA-mRNA/hub genes network. Circular glutamine and Serine rich 1 (circQSER1), circular formin 2 (circFMN2), and circular coiled-coil Domain containing 7 (circCCDC7) were selected to construct the network

**Fig. 7:**
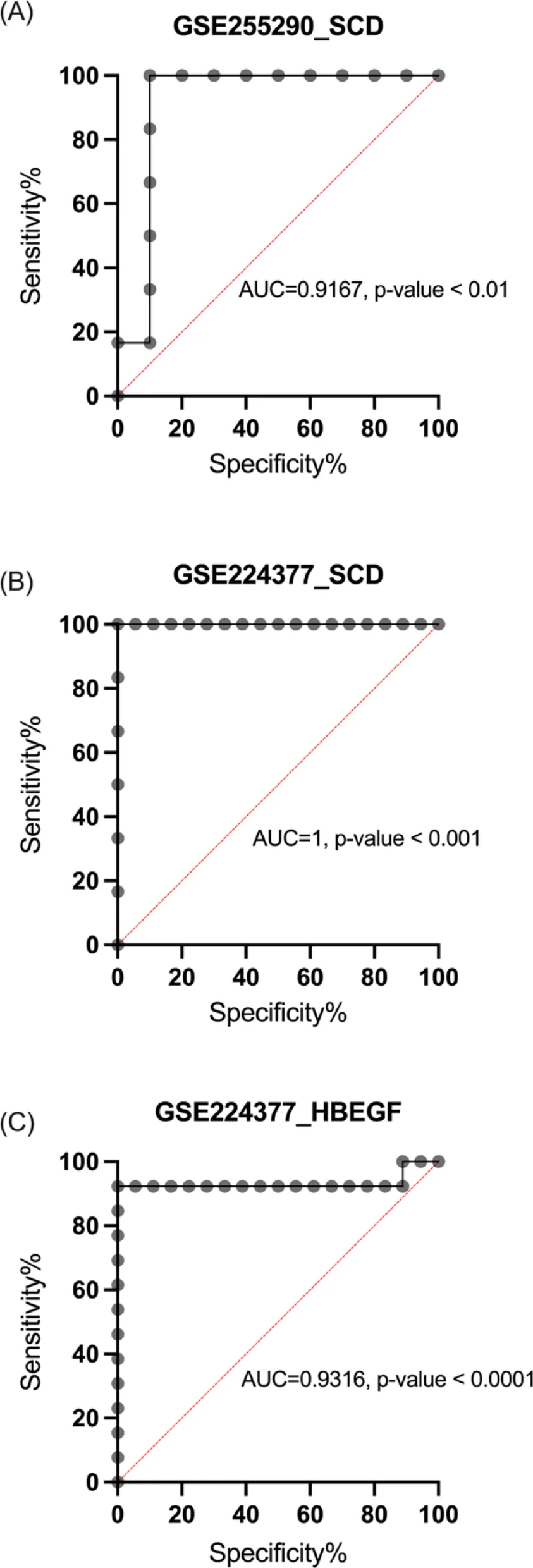
ROC curves of the hub genes **A** GSE255290_SCD,**B** GSE224377_SCD and **C** GSE224377_HBEGF.Each panel shows sensitivity against specificity, and the red dashed diagonal line representing the chance performance. The AUC and p-value were reported as accuracy and statistical significance

To further explore the regulatory functions of circRNAs in MS, miRNAs which target both DE circRNAs and DE mRNAs within the specific lesion type were predicted (Table S2–S15). The significant networks were constructed and shown in Fig. 6. circQSER1 and circFMN2 that targeted *SCD* via hsa-miR-6891-5p, hsa-miR-6758-5p, and hsa-miR-4728-5p were down-regulated in AL type. circCCDC7/hsa-miR-4659b-3p/*HBEGF* network was down-regulated in CA type (Fig. 6). White matter-specific expression of the miRNAs was assessed using miRNATIssueAtlas 2025. All four miRNAs that were involved in the potential networks were detectable in human brain tissue, eventually confirming these miRNA loci were transcriptionally active in the relevant brain tissue compartment (Table 1). Among the circRNA-miRNA-mRNA pairs, *SCD* and *HBEGF* were identified as hub genes based on the high confidence of PPI network and score of network connectivity (Table 2). The consistency of *SCD* and *HBEGF* expression was assessed from the validation of GEO database (Fig. 7). *SCD* expression demonstrated strong performance in GSE255290 (AUC (area under the curve) = 0.9167) and GSE224377 (AUC = 1) with statistically significant p-value. *HBEGF* was not present in the GSE255290 dataset, but was validated in GSE224377 (AUC = 0.9316, p-value < 0.0001) that revealed the high performance with strong statistical significance of this association in MS samples.

**Table 1:** Expression of miRNAs in white matter of the brain region

miRNA_id	avg_expression	stddev_exprression
hsa-miR-6891-5p	0.0492	0.2276
hsa-miR-6758-5p	0.4791	4.6533
hsa-miR-4728-5p	13.1724	80.0214
hsa-miR-4659b-3p	0.0358	0.1139

**Table 2:** Hub genes that were involved in the circRNA-miRNA-hub genes network

Stringdb identifier	Gene name^1^	Score_MCC	Score_MNC
9606.ENSP00000359380	*SCD*	1	1
9606.ENSP00000230990	*HBEGF*	2	0.31

^1^stearoyl-CoA (*SCD*), herapin-binding EGF like growth factor (*HBEGF*)

**Fig. 8:**
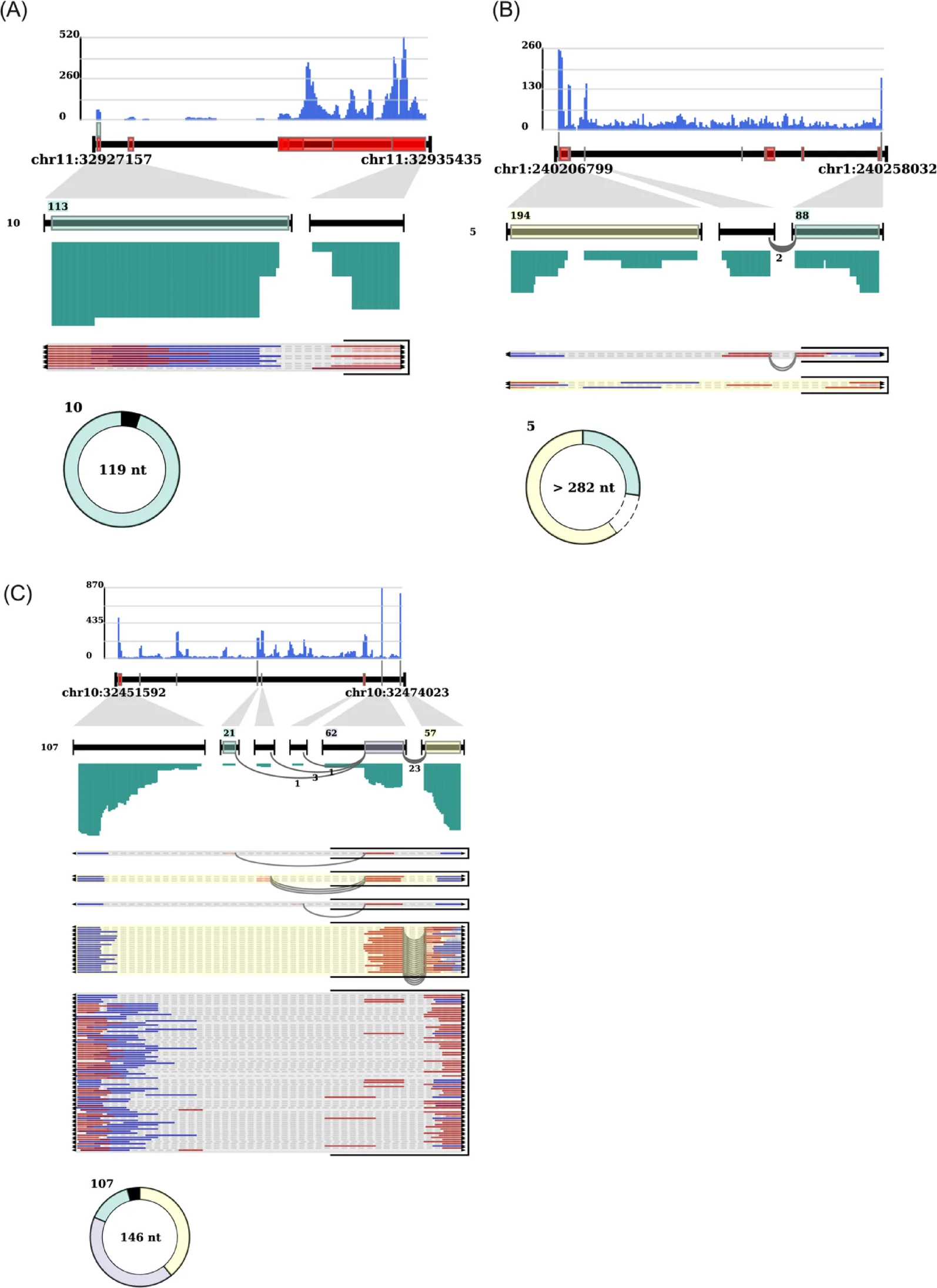
Visualisation of the circRNAs **A** circQSER1 (chr11:32927157–32935435), **B** circFMN2 (chr1:240206799–240258032) and **C** circCCDC7 (chr10:32451592–32474023). For each circRNA, the top track displayed RNA-seq coverage across the genomic locus. Annotated exons involved in circularisation were shown as blue colour along the genomic coordinate bar. BSJs were indicated by curved arcs, with the number on the left denoting the supporting junction read counts. Horizontal bar plots below illustrated the distribution and abundance of junction-spanning reads. Linear read alignments were shown to highlight reads supporting both canonical splicing and back-splicing events. The reconstructed circRNAs were shown at the bottom of each panel which summarised the exon composition and transcript length

The visualisation of one of isoforms for each circQSER1 (chr11:32927157–32935435), circFMN2 (chr1:240206799–240258032) and circCCDC7 (chr10:32451592–32474023) is displayed as various circRNAs isoforms across their exhibiting lesion types and their internal splice events (Fig. 8). All isoforms were shown in Fig. S4. RNA-seq coverage was enriched over circRNA-forming exons, indicating active transcription at these loci. Clear backsplice junctions (BSJs) for each circRNAs were detected, supported by multiple junction-spanning reads.

## Discussion

Secondary progression multiple sclerosis (SPMS) is characterised by pathological heterogeneity, with several lesion types reflecting distinct mechanisms of disease progression. While the majority of work has entailed transcriptional changes at the mRNA level, the contribution of circRNAs to lesion-specific molecular regulation remains unexplored. In this study, we performed a comprehensive lesion-specific reanalysis of the publicly available RNA-seq dataset to investigate the circRNA and mRNA expression as well as circRNA-miRNA-mRNA/hub genes networks across NAWM, AL, CA, IL and control white matter.

DE analysis on the circRNAs and linear transcripts displayed lesion-dependent expression patterns across MS pathology (Figs. 1, 2 and 3). Across the circRNA profiling, extensive down-regulated circRNAs pattern was evident across all lesion types, which closely aligned with a previous study by Iparraguirre and Muñoz-Culla [24] A moderate number of DE circRNAs and a comparably low number of DE linear transcripts detected from NAWM was indicative of SPMS progression with mild biological abnormality occurring. As the disease progressed to AL, there were pronounced transcriptional shifts including more extensive dysregulation of both circRNAs and linear transcripts. The transcriptional alterations at AL stage reflected the worse chain reactions occurring during the robust inflammation, demyelination, and neuronal disruption. CA and IL had less DE circRNAs and linear transcripts comparing to AL, but ongoing activation of inflammatory responses and tissue damage still highlighted their transcriptional difference when comparing to the normal tissue. Overall, a larger number of down-regulated RNA expression in comparison to up-regulated RNA expression was observed consistently across disease samples. This can be explained through smouldering inflammation and immune activity at the lesions, resulting in cell death.

Various studies have demonstrated the complexity of MS pathology aligning with both neurological and autoimmune processes [3, 56]. Current enrichment analysis has been built based on the protein functions with the host gene names, yet the circRNAs functions have not been widely annotated. Hence the enrichment analysis in the study provided an overview of the biological roles of circRNAs by functionally annotating their host genes. The genes of the dysregulated circRNAs from AL lesion type of SPMS enriched key GO terms and KEGG pathways reinforced the focus on immune system activation, inflammatory responses, and neurodegeneration (Fig. 4). Biological enrichment analysis in this study revealed that multiple signalling pathways including GTPase, mitogen-activated protein kinase (MAPK), forkhead ox O (FoxO), and cyclic AMP (cAMP) which are known for regulating T- and B-cell activation and neuroinflammation in MS, has also been mentioned in the previous literatures [57–60]. Epidermal growth factor and ErbB receptor signalling were enriched by the down-regulated circRNAs, which signify the disruption of remyelination and oligodendrocytes formation [61, 62]. Several enriched terms were associated with apicobasal polarity and adherens junctions. Adherens junctions are adhesion complexes, primarily consist of cadherin-catenin proteins, that can function as connecting the synapses and maintaining structural integrity. Dysregulated adherens junctions can worsen the apicobasal polarity and further damaging the blood-brain barrier in MS patients [63–65]. Dysfunction of apicobasal polarity in the brain endothelial cells can lead to leakage of the blood-brain barrier [63]. Synaptic pathology was widely enriched due to the extensive synaptic loss and neuroinflammation, particularly the loss of pre-synaptic terminals in demyelinated areas and the deconstruction of post-synapse density and dendritic spines [66]. Up-regulated circRNAs were mapped to epigenetic repression, kinase signalling, and chromatin remodelling [67]. Serine/threonine kinases have been identified as key regulators for inflammation, promoting Th17 cell differentiation, and progressive demyelination [68]. The imitation switch (ISWI), an ATP-dependent chromatin remodelling complex was the only statistically significantly enriched in the CC from the up-regulated circRNAs. It has been found that ISWI complexes regulate the oligodendrocyte precursor cells and oligodendrocyte differentiation [69]. Therefore, we hypothesise that the correlation with ISWI complexes and oligodendrocytes may play significant regulatory roles in remyelination in SPMS. The enrichment of terms consistently displayed close relationships with synapses, cellular structure, blood-brain barrier, immune systems, and epigenetics, highlighting a cascade of reactions including the synaptic dysfunction, cytoskeletal instability, disruptions across immune systems and epigenetics driving the disease progression.

To explore the regulatory potential of circRNAs, we constructed circRNA-miRNA-mRNA networks for all four lesions. Hub genes were selected for circRNA-miRNA-hub genes interactions from the protein-protein interaction (PPI) networks based on their degree of connectivity (Fig. 6; Table 2). Our analysis identified networks including circQSER1-hsa-miR-6891-5p/hsa-miR-6758-5p-*SCD*, circFMN2-hsa-miR-4728-5p-*SCD*, circCCDC7-hsa-miR-4659b-3p-*HBEGF*. Of note, expression of the predicted miRNAs in human brain tissue were analysed by miRNATissueAtlas 2025 through computing the mean reads per million mapped reads (avg_expression) across multiple biological replicates from independent GEO studies labelled as “brain” [53]. The miRNAs were lowly expressed in human brain, indicating their potential biological function within the ceRNA in SPMS. While human circQSER1 remains unexplored, this study revealed the circularisation of *QSER1* and its diverse isoforms across all identified AL samples. The parent gene of circQSER1 has been found to protect DNA methylation process from hypermethylation [70]. Linear QSER1 was found to regulate circRNAs derived from presenilin-1 gene (*PSEN1*), sponging the miRNAs (hsa-mir-4668-5p and hsa-mir-5584-5p) in Alzheimer’s disease [71]. Both Alzheimer’s disease and MS are neurological diseases which exhibit widespread DNA methylation changes to alter gene expression, resulting in pathogenesis [72, 73]. Therefore, down-regulation of circQSER1 expression may indicate the potential effect and mechanisms in this study. miRNAs (hsa-miR-6891-5p, hsa-miR-6758-5p) that interacted with circQSER1 and *SCD* have been previously identified to regulate RNA molecules in intrahepatic cholestasis of pregnancy and gastric cancer, respectively [74, 75]. Given the small cohort size of GEO dataset (*n* = 10), AUC = 1.0 from *SCD* gene was reported as exploratory observation using independent MS-derived dataset (Fig. 7). Consistent expression trends and strong discriminative performance in the analysis of *SCD* showed robustness of the hub gene in MS [76]. CircFMN2 (hsa_circ_0005100), which is widely discovered in prostate cancer and colorectal carcinoma, plays a role in sponging other miRNAs that regulate downstream mRNAs and protein expression that impacts severely on tumorigenesis and metastasis [77, 78]. Previous cohort studies revealed and discussed the potential decreased risk of prostate cancer and colorectal cancer in the presence of MS, however, the variance across different studies require further validation [79]. Our results showed the down-regulation of circFMN2 in MS, which has been found to promote carcinoma in prostate and colorectal cancer. Overall, more cohort studies and experiments are necessary to understand the relationship between circFMN2 and different diseases.

CircCCDC7, which was down-regulated in the CA lesion type in our analysis (Fig. 6), has also been reported in a prostate cancer study [80]. Chen and Wang [80] established that circCCDC7 was a prognostic marker since circCCDC7 was reduced significantly in prostate cancer and its overexpression led to cell apoptosis. At CA lesion, we predicted that circCCDC7 acted as hsa-miR-4659b-3p sponge to regulate *HBEGF* expression. Previous studies have identified that *HBEGF* induced more at the early stage of MS for inducing protective mechanisms, including remyelination [61, 81]. Remyelination in the inflamed central nervous system is driven predominantly by newly differentiating oligodendrocyte precursor cells (OPCs) [82]. The epidermal growth factor signalling pathways promote the recruitment, proliferation, and migration of OPCs to the demyelinated lesions [61]. Linnerbauer and Lößlein [61] explicitly stated that the up-regulation of *HBEGF* at the early stage of MS was induced by the astrocytes to protect against neuroinflammation, followed by reduction in expression in the later stages. Their discovery closely aligned to our findings as *HBEGF* decreased at chronic active lesion type of SPMS, which is characterised by persistent inflammation within the central nervous system and ongoing demyelination. The ROC validation result (Fig. 7) also strengthen the translational relevance of our finding of *HBEGF* and suggests that circRNA-associated expression have utility beyond the discovery cohort.

From the circRNAs visualisation, read coverage profiles supported robust BSJ signals, and the alignment of circRNA junction reads with annotated exons confirmed canonical exon-derived circularisation instead of artefactual splicing [54]. The isoform profiles for circQSER1 and circFMN2 (Fig. 8, Fig. S4) were predominantly observed in AL lesion. AL stage represents an early and highly inflammatory stage, and the limited number of circRNA isoforms at this stage may reflect a tighter splicing mechanism (Fig. 1) [83]. While the circRNA identification analysis in this study revealed greater number of circRNAs in CA stage comparing to AL, the extensive 14 isoforms of circCCDC7 in CA stage was particularly notable (Fig. S4). CircCCDC7 harboured the greatest number of junction read counts, potentially indicating its extensive expression through alternative circularisation.

Since our study was a bioinformatic analysis of bulk RNA-seq data, several limitations can be addressed. The sample of origin was retrieved from human post-mortem brain tissue. Although Elkjaer and Frisch [2] classified the lesions in their Methods, the real-time dynamic shift of the DE circRNAs and mRNAs failed to be monitored across the SPMS lesions. Although the dataset provided valuable resolution across different MS lesion types, the sample size within each lesion type after stringent filtration remains limited. In bioinformatics analysis of RNA-seq data, sequence quality control is a prerequisite step to minimise false-positive signals arising from low-complexity reads, adapter contamination, and poor alignment rates [31, 33, 84]. Thus, to overcome the introduction of systematic noise into the DE analysis and network prediction, we reduced the samples to maintain reliability. The resulted AL stage represented the principal constraint on the certainty of conclusions. Particularly, remyelinating lesion was excluded after the dataset quality selection step, which limited the discovery and comparison of lesion-specific circRNA-associated analysis. Increasing the number of samples per lesion type with dedicated quality controlled sequencing profiling would improve statistical power and enable more refined circRNA expression across lesions. This bulk RNA-seq data that captured average transcript abundance across heterogenous cell population within each lesion, however, MS pathogenesis is usually accompanied by changes on diverse cell types, including oligodendrocytes, astrocytes, microglia, infiltrating immune cells, and neurons [85–88]. Hence, circRNA expression patterns and isoforms cannot be identified for their attribution in specific cell types. The integration of single-cell or single nucleus RNA-seq would help to depict cell-type circRNA profiling [85, 89–92]. Although advanced algorithms like CIRI offers sophisticated statistical models to provide more accurate expression values, long-read sequencing technologies can characterise full-length circRNA isoforms with higher confidence and validate the identified isoforms [93]. Moreover, the circRNA-miRNA-mRNA/hub genes networks were constructed based on computational predictions and multiple databases. Functional validation will be essential to establish the biological relevance of key findings in this study [94]. Experimental approaches such as circRNA knockdown or overexpression, quantitative real-time PCR (qRT-PCR) and western blot can validate the interactions and verify gene expression under microarray analysis [95, 96]. Comparison of circRNA isoform diversity across lesion types in SPMS was confounded by variability in back-splice junction read depth between groups, and conclusion about lesion-specific alternative back-splicing can only be drawn when additional expression is performed on the identified circRNA isoforms. Integrating multi-omics approaches can provide a more comprehensive perspective of circRNA function and regulatory roles for inflammation, neurodegeneration, immune responses in MS progression [97, 98]. Independent validation through Mendelian randomisation leveraging genome-wide association data would further strengthen the evidence for the candidate hub genes identified in this study [99, 100]. Furthermore, reliable molecular discovery in complex SPMS typically required replication across cohorts of substantially larger size in order to progress toward diagnostic application [101].

A limitation of the present study is that the analyses were performed using bulk RNA-sequencing data from heterogeneous white matter lesions. Future studies integrating circRNA profiling with single-cell or single-nucleus transcriptomic datasets, or applying reference-based deconvolution approaches to bulk RNA-seq data, will be valuable for disentangling cell-type composition effects from cell-intrinsic regulatory changes and for refining the cellular context of lesion-specific circRNA dysregulation.

In conclusion, we established a comprehensive transcriptome-wide analysis across SPMS lesion types in this study. The profiling revealed that the expression of circRNAs and linear transcripts were highly lesion-specific and closely associated with pathology. By constructing circRNA-miRNA-mRNA/hub genes networks, we identified several key circRNAs including circCCDC7, circFMN2, and circQSER1 which contributed to lesion-specific regulatory capacity including binding to specific miRNAs and targeting *SCD* and *HBEGF* expression within the lesion type. The finding provided a resource for future functional investigation and potentially served as biomarkers in for future biological studies in MS disease.

## Supplementary Information

Below is the link to the electronic supplementary material.

Supplementary Material 1

Supplementary Material 2

## Data Availability

All data supporting the findings of this study are available within the paper and its Supplementary Information.
