# Supplementary material for: Lesion-specific circular RNA and mRNA signatures in secondary progressive multiple sclerosis
Source: Mol Brain. 2026 Jul 22;19:71. doi: 10.1186/s13041-026-01333-y (PMC13679284; doi:10.1186/s13041-026-01333-y)
Supplement: Supplementary file 1 [file 13041_2026_1333_MOESM2_ESM.docx]

**Lesion-specific circular RNA and mRNA signatures in secondary progressive multiple sclerosis**

Yulan Gao1, Konii Takenaka1, Kristina Santucci1, Grace Lindner1, Yuning Cheng1, Si-Mei Xu1, Michael Janitz1*

1 School of Biotechnology and Biomolecular Sciences, University of New South Wales, Sydney, NSW 2052, Australia

* Correspondence: m.janitz@unsw.edu.au

Supplementary figures

[Figure S1. GO terms and KEGG pathways analysis for NAWM.	3](#_Toc223617155)

[Figure S2. GO terms and KEGG pathways analysis for IL.	5](#_Toc223617156)

[Figure S3. GO terms for CA.	7](#_Toc223617157)

[Figure S4. Visualisation of circRNA isoforms.	11](#_Toc223617158)

Figure S1. GO terms and KEGG pathways analysis for NAWM. (A – B) MF and KEGG terms enriched by down-regulated circRNAs. (C – D) BP and CC terms enriched by up-regulated circRNAs.

Figure S2. GO terms and KEGG pathways analysis for IL. (A - D) BP, CC, MF, and KEGG terms enriched by down-regulated circRNAs. (E – F) MF and KEGG terms enriched by up-regulated circRNAs.

Figure S3. GO terms for CA. (A - C) BP, CC, and MF terms enriched by down-regulated circRNAs. (D) BP terms enriched by up-regulated circRNAs.

Figure S4. Visualisation of circRNA isoforms. (A) circQSER1 (chr11:32927157-32935435), (B) circFMN2 (chr1:240206799-240258032) and (C) circCCDC7 (chr10:32451592-32474023).
